# First report of the pyrethroid-resistance-associated *V1016I* mutation in *Aedes aegypti* at the seaport of Cotonou, Benin, West Africa

**DOI:** 10.1186/s41182-025-00806-5

**Published:** 2025-09-30

**Authors:** Antoine Salomon Lokossou, Rock Aikpon, Murielle Dossou, Bruno Adjottin, Fridolin Ubald Dossou-Sognon, Alphonse Konkon, Halid Bakary, Halalou Ali Mamam, Yao Abotsi, Erick Akpo, Richard Akanni-Ediko, Anges Yadouleton

**Affiliations:** 1 Plateforme Portuaire de Surveillance Environnementale (PPSE), BP 927, Cotonou, Benin; 2 Université Nationale des Sciences Technologies, Ingénierie et Mathématiques (UNSTIM), BP 2282 Goho, Abomey, Benin; 3 Centre de Recherche Entomologique de Cotonou (CREC), 06 BP 2604 Cotonou, Benin; 4 University of Abomey -Calavi (UAC), Abomey-Calavi, Benin

**Keywords:** Insecticide resistance, *Anopheles gambiae*, *Aedes aegypti*, *V1016I mutation*, *Kdr* mutation

## Abstract

**Background:**

Monitoring insecticide resistance in disease vectors is a key strategy to anticipate emerging and re-emerging diseases, particularly in the context of interventions such as vector control interventions. This study aims to assess the insecticide resistance profile of three major mosquito species in the Port of Cotonou and to characterize the molecular mechanisms associated with resistance, with a focus on mutations in *Aedes aegypti* and *Anopheles gambiae* s.l.

**Methods:**

This study was conducted at the Port of Cotonou from June 2023 to November 2024. Standard WHO susceptibility tube tests were performed using several insecticides: deltamethrin, permethrin, and alpha-cypermethrin (pyrethroids), bendiocarb (carbamate), and pirimiphos-methyl (organophosphate). The potential role of metabolic resistance mechanisms was investigated through synergist assays using piperonyl butoxide (PBO). In addition, molecular analyses were performed: SINE-PCR was used to differentiate *Anopheles gambiae* from *Anopheles coluzzii*, while allele-specific PCR (AS-PCR) assays were employed to detect the mutations *L1014F* and *L1014S* in *Anopheles gambiae s.l*., as well as the *F1534C, S989P*, and *V1016I* mutations in *Aedes aegypti*.

**Results:**

The three mosquito species tested showed confirmed resistance to most pyrethroids (rate mortality < 90%). The addition of PBO significantly restored the efficacy of alpha-cypermethrin, with mortality rates of 93% in *Culex*, 100% in *Aedes*, and 98.97% in *Anopheles*, compared to 45, 89.80, and 69.39% respectively, when alpha-cypermethrin was used alone. This difference was statistically significant, with *p*-values < 0.05. Pirimiphos-methyl remained effective, inducing 100% mortality across all species. Suspected resistance to bendiocarb was observed in *Aedes aegypti* and *Anopheles gambiae*, with respective mortality rates of 92.78 and 95.83%, while resistance to bendiocarb was confirmed in *Culex quinquefasciatus*, with a mortality rate of 85.86%.

The analysis of *kdr* mutations in *Anopheles gambiae s.s.* and *Anopheles coluzzii* revealed a high prevalence of the *L1014F* mutation in both species, with allele frequencies of *f*(*F*) = 0.8421 in *An. gambiae s.s.* and *f*(*F*) = 0.9292 in *An. coluzzii*. No individuals carried the wild-type homozygous genotype (SS) for this mutation, indicating a high level of fixation of the resistant *L1014F* allele, particularly in *An. coluzzii*.

The *L1014S* mutation, previously detected in northern Benin, was identified for the first time in the southern region in heterozygous *Anopheles gambiae s.s.* individuals. This mutation was absent in *An. coluzzii* but was detected at a very low frequency of *f*(*S*) = 0.052 in *An. gambiae s.s.*. For the *F1534C* mutation, the frequency of the mutant C allele was *f*(*C*) = 0.7284. Regarding the *V1016I* mutation, the mutant I allele had a frequency of *f*(*I*) = 0.1935. Notably, this is the first detection of this mutation in Benin.

Finally, the *S989P* mutation showed an allele frequency of *f*(*P*) = 0.637 for the mutant P allele. These findings reveal a high prevalence of the *F1534C* and *S989P* mutations, while the *V1016I* mutation appears at a more moderate frequency in the tested population.

**Conclusion:**

This study confirms widespread resistance to pyrethroids among major mosquito vectors in the Port of Cotonou. The detection of *L1014S* and *V1016I* mutations highlights an evolving dynamic of *kdr*-mediated resistance. These findings underscore the urgent need for strengthened insecticide resistance monitoring within an integrated vector management framework, taking into account the target species, underlying resistance mechanisms, and local ecological contexts.

## Introduction

Biological invasions represent a major public health concern for all countries, as they pose a significant threat to biodiversity and ecosystems on a global scale. [1]. As of 2023, more than 37,000 alien species have been recorded globally, of which at least 3500 are classified as invasive species [2]. The global cost of managing invasive alien species was estimated at 162.7 billion USD in 2017 [3]. Several factors contribute to this phenomenon, but the crossing of geographical barriers largely driven by human activities remains the primary cause [4].

Among invasive alien species in the animal kingdom, insects are the most represented. Some of them are rapidly expanding their geographical range worldwide, such as *Aedes albopictus* and *Anopheles stephensi*, two species originally from Asia. These two species are responsible for the transmission of several emerging or re-emerging diseases, such as malaria, dengue, chikungunya, and Zika, in areas where these diseases were previously absent [5, 6].

The fight against biological invasions relies on several key pillars: surveillance, detection, and control. In the absence of effective vaccines for most vector-borne diseases, many of which are transmitted by mosquitoes such as *Anopheles*, *Aedes*, and *Culex*, vector control strategies, particularly the use of insecticides, remain the primary and most widely used means of prevention globally [7]. However, their effectiveness is increasingly compromised by the emergence of insecticide resistance in vector populations [8], as well as by the exophilic and diurnal behavior of species such as *Aedes aegypti*, which escape conventional interventions like indoor residual spraying and insecticide-treated nets [9]. These insecticides belong to various chemical classes, including carbamates, organophosphates, organochlorines, pyrethroids, and more recently, pyrroles and neonicotinoids. However, these classes differ in their modes of action and therefore do not target the same physiological sites. The pyrethroid class has remained the most widely used over the past decades. It is primarily employed for insecticide-treated nets (ITNs), indoor residual spraying (IRS), and offers the advantage of low toxicity to mammals.

However, the effectiveness of these compounds is increasingly compromised by the emergence of resistance mechanisms developed by mosquitoes [10]. These resistance mechanisms notably involve target-site mutations, particularly the knockdown resistance gene, which enables mosquitoes to tolerate and survive insecticide exposure. This resistance, known as knockdown resistance (*kdr*), induces cross-resistance to pyrethroids and DDT in several mosquito species, including *Aedes* spp. and *Anopheles *spp. [11–13]. This poses a significant threat to vector control programs that rely on the use of these compounds.

Two mutations have been identified in *Anopheles gambiae* s.l., the major malaria vector in Africa: the *L1014F* mutation and the *L1014S* mutation [13]. In *Aedes aegypti*, several mutations have been identified in the voltage-gated sodium channel, including *V1016I*, *V1016G, F1534C,* and *S989P*, which are associated with resistance to pyrethroid insecticides. The present study aims to assess the susceptibility of major vectors to different classes of insecticides, to highlight the *L1014F* and *L1014S* mutations in *Anopheles gambiae* s.l., and the *V1016I, S989P,* and *F1534C* mutations in *Aedes spp.,* and finally to estimate the allele frequencies of these mutations within the studied populations. The Port of Cotonou, the main maritime gateway of Benin and one of the busiest in West Africa, represents a high-risk zone for the introduction and spread of various vectors and their pathogens. This vulnerability is exacerbated by the intensity of international goods traffic and environmental conditions favorable to mosquito proliferation. In line with World Health Organization (WHO) recommendations, rigorous surveillance at such points of entry is essential to prevent and respond promptly to vector invasions. In this context, the selection of the Port of Cotonou as a strategic study site for entomological surveillance and insecticide resistance management is fully justified.

## Methods

### Study area

**Fig. 1:**
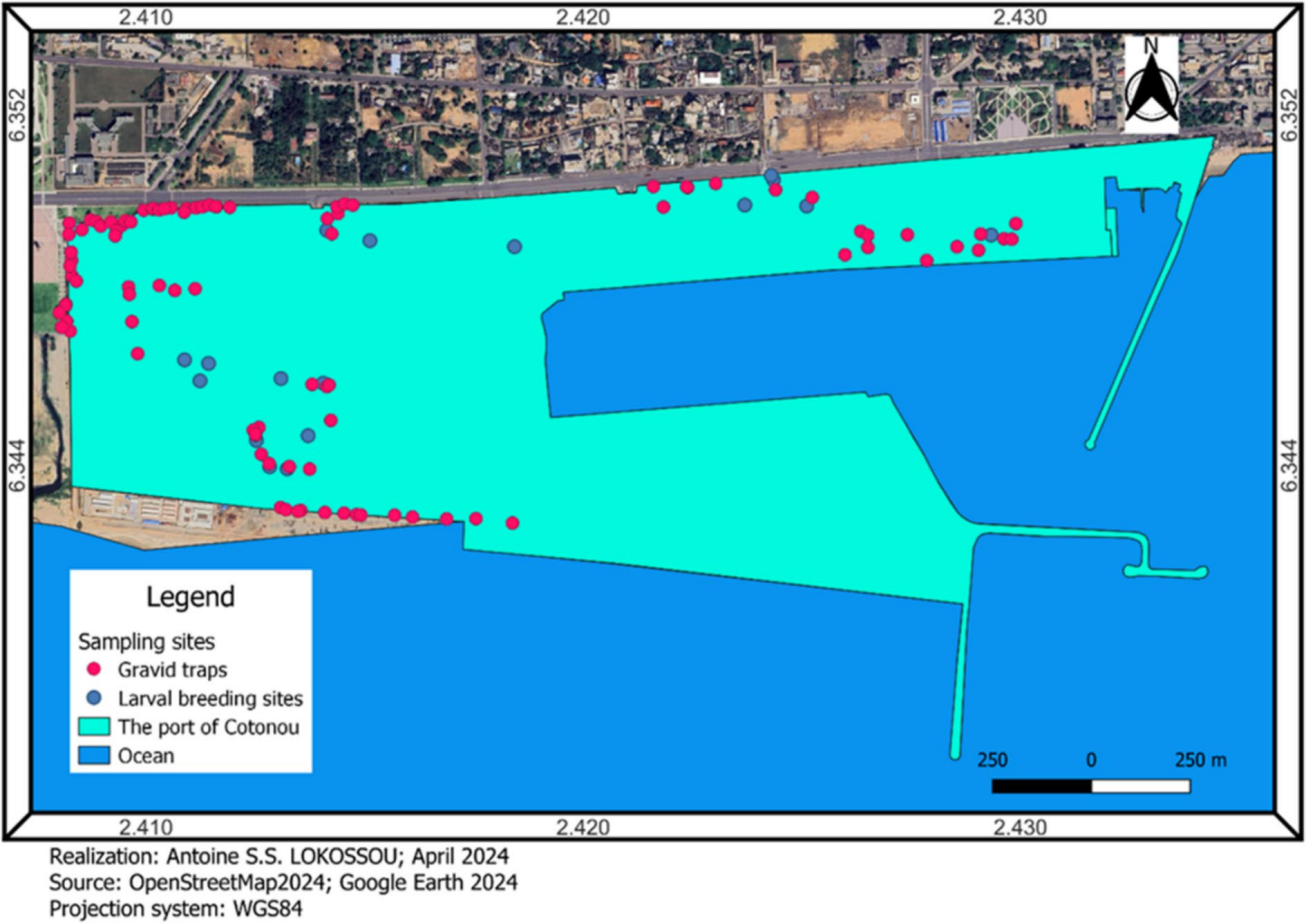
Port of Cotonou with the different sampling sites

Larval collection was conducted from June to November 2023 and was part of a larger study carried out from June 2023 to May 2024 at the Port of Cotonou. The port is located in the city of Cotonou, the economic capital of Benin, covering an area of approximately 79 km^2^ (Fig. 1). It is the country’s main administrative, commercial, and industrial center. The city has a subequatorial climate, characterized by two rainy seasons (from March to July and from September to November) and two dry seasons (from November to February and from July to August), with annual rainfall ranging between 1200 and 1500 mm.

### Larval collection methods

Larval collection for the various tests in this study was carried out over a one-year period, from June 2023 to November 2024, using two distinct methods: direct collection of larvae of different species from various potential breeding sites, and larval collection and ovitrap use.

### Larval collection from breeding sites

**Fig. 2:**
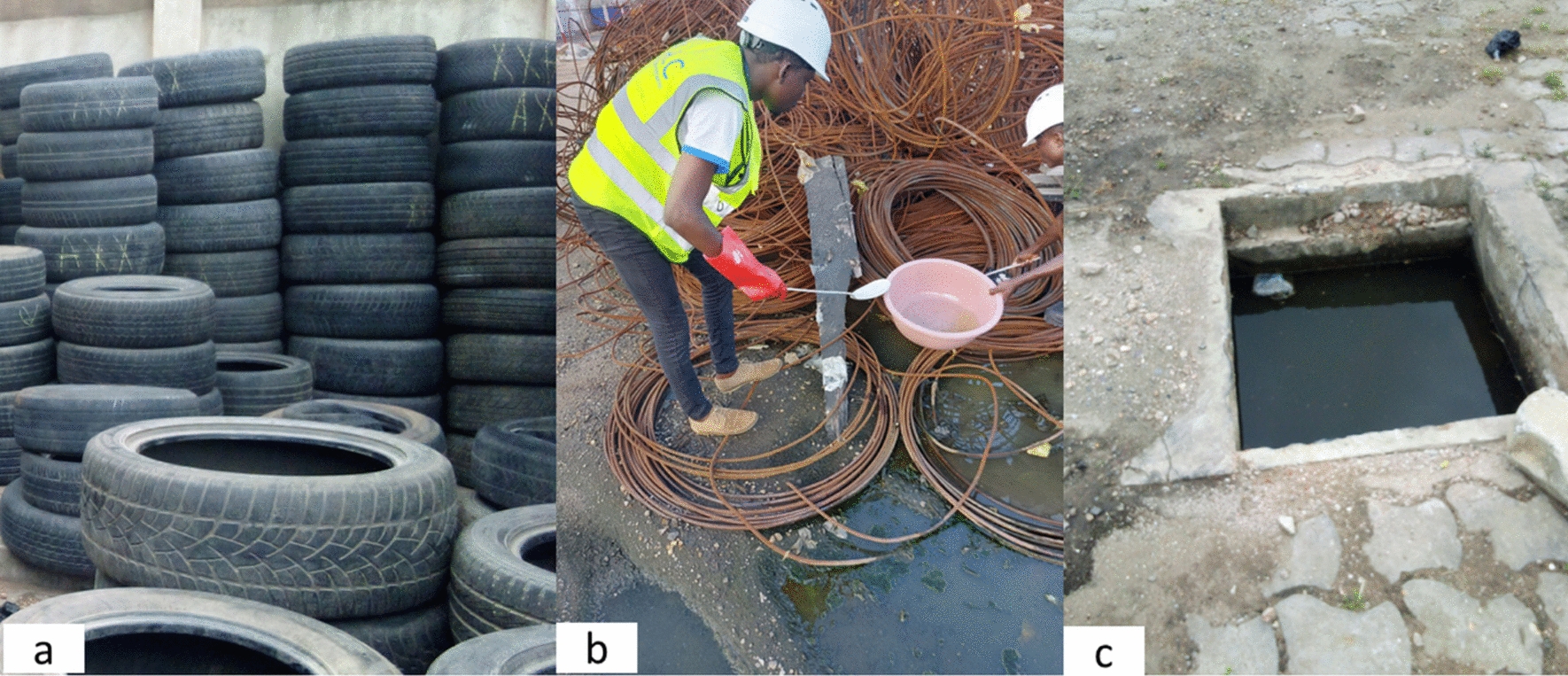
Different larval breeding sites sampled: **a** *Aedes* breeding site; **b** *Anopheles* breeding site; **c** *Culex* breeding site

Larvae and nymphs are collected directly from breeding sites such as tires (Fig. 2a), water puddles (Fig. 2b), gutters (Fig. 2c), and cans, all of which are georeferenced (Fig. 1). The dipping method is used for the collection of *Anopheles* and *Culex* [14]. For *Aedes*, the entire contents of the breeding site are transferred into a container for observation and sorting in the laboratory. These larvae and nymphs are then filtered, stored in labeled jars, and transported to the insectarium for breeding.

### Larval collection and use of ovitraps

**Fig. 3:**
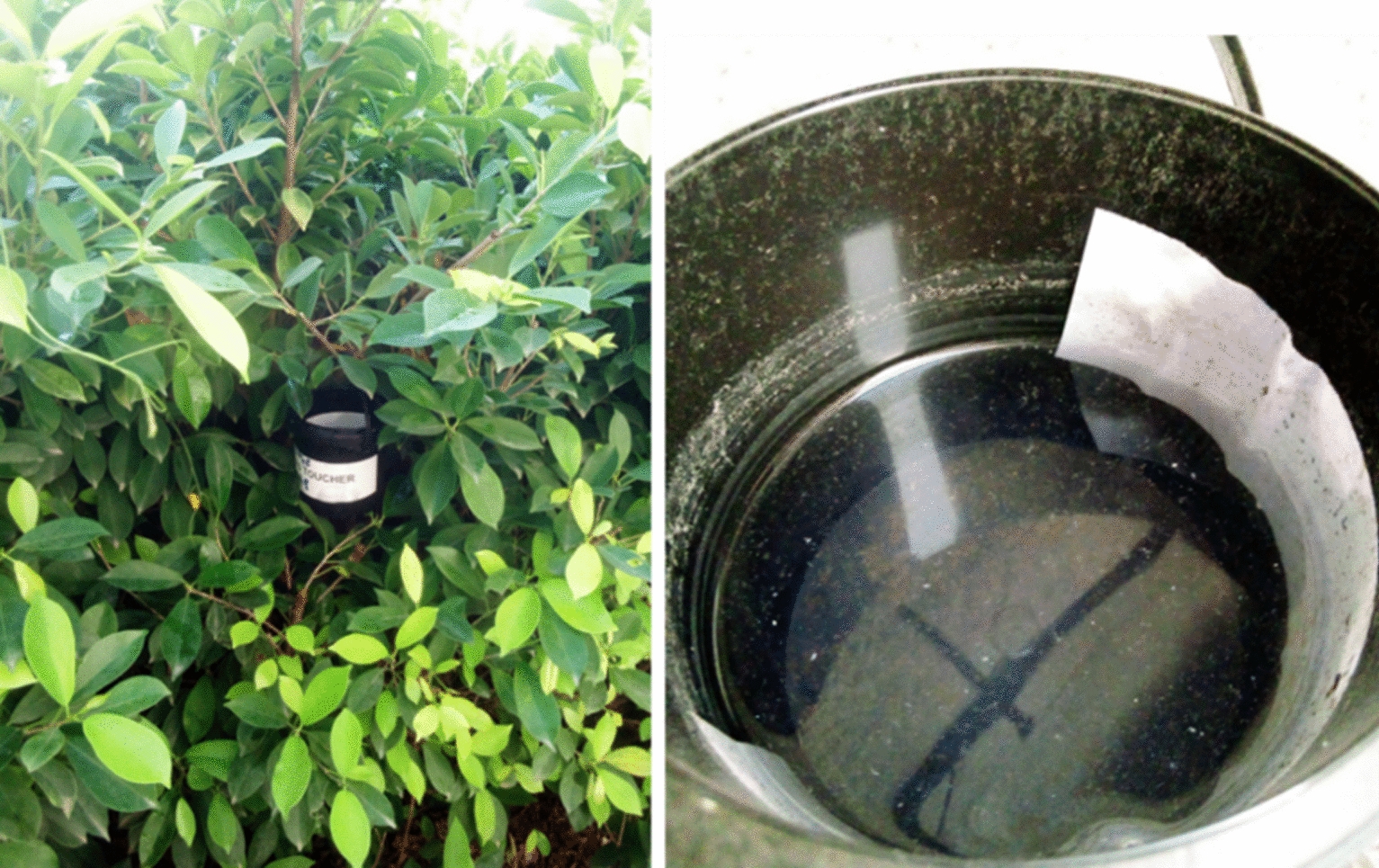
Oviposition trap for collecting *Aedes* larvae

Oviposition traps are devices used for the collection of mosquito larvae and nymphs, particularly from the genus *Aedes*. We used plastic containers, preferably black in color, filled with water up to two-thirds of their capacity [15]. Each trap is equipped with either a white paper support placed on the internal walls (Fig. 3) or a submerged piece of wood, serving as an oviposition site. Attracted by the stagnant water, female mosquitoes come to deposit their eggs on these structures. The traps are regularly inspected until larvae and/or nymphs are observed, which are then collected and reared in the laboratory for further analysis.

### WHO-tube susceptibility test

**Fig. 4:**
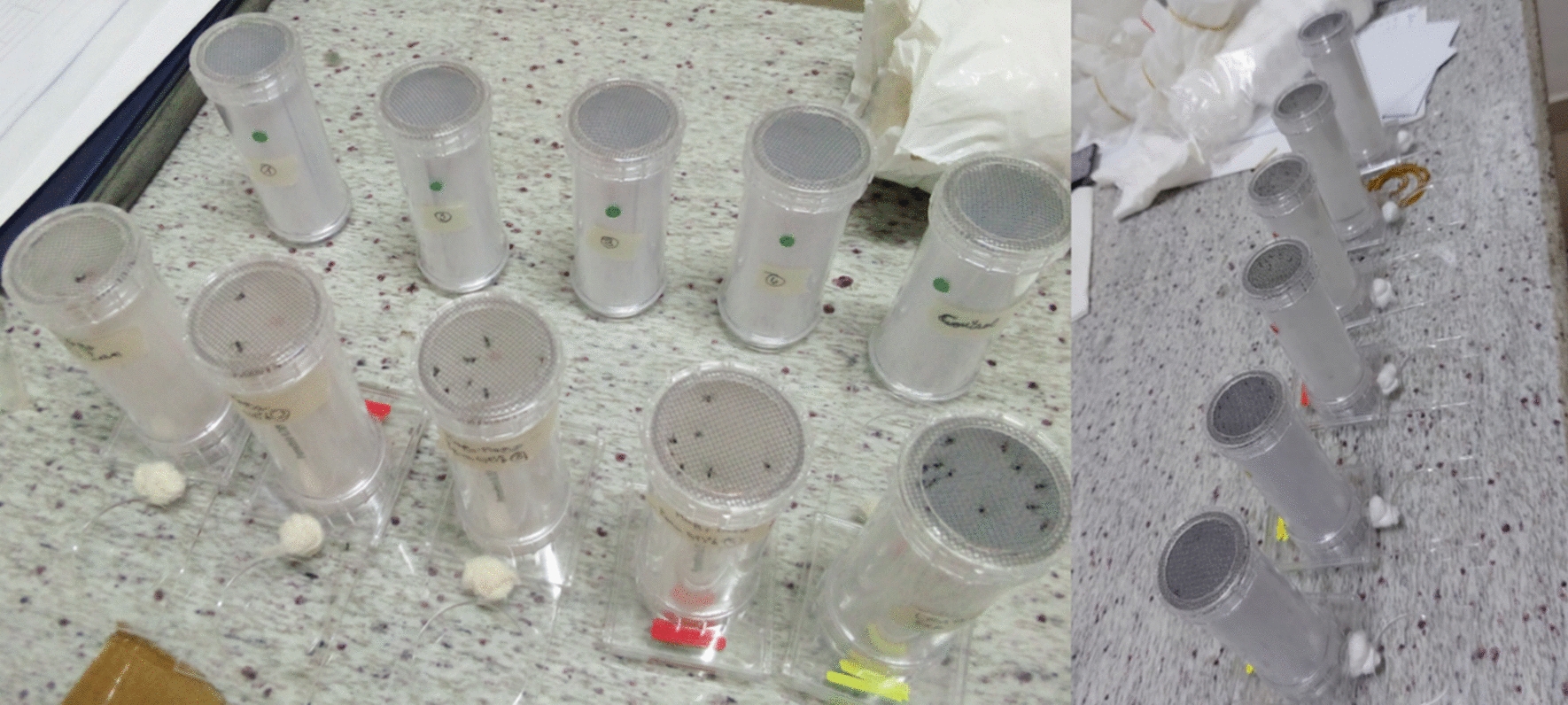
WHO tube for susceptibility tests

Female mosquitoes of the genera *Aedes, Culex, and Anopheles*, obtained from larvae collected in the field, were used for susceptibility tests in tubes according to the WHO protocol [16]. Only F1 females, unfed and aged 3 to 5 days, were exposed to several insecticide molecules (Table 1), because only females are hematophagous, act as vectors, and live longer than males (WHO, 2022) (Fig. 4).

**Table 1:** Types of insecticide molecules employed

Species	Insecticides tested
*Aedes*	Bendiocarb 0.2%
	Pirimiphos methyl 60 mg/m^2^
	Deltamethrin 0.03%
	Permethrin 0.4%
	Alpha-cypermethrin 0.05%
	Alpha-cypermethrin 0.05% + PBO
*Culex*	Bendiocarb 0.1%
	Pirimiphos methyl 0.25%
	Deltamethrin 0.025%
	Permethrin 0.25%
	Alpha-cypermethrin 0.05%
	Alpha-cypermethrin 0.05% + PBO
***Anopheles***	Bendiocarb 0.1%
	Pirimiphos methyl 100 mg/m^2^
	Deltamethrin 0.05%
	Permethrin 0.75%
	Alphacypermethrin 0.05%
	Alphacypermethrin 0.05% + PBO

The different insecticide-impregnated papers used for the tests were ordered from the Vector Control Research Unit, School of Biological Sciences, Universiti Sains Malaysia.

For each mosquito species tested, batches of 20 to 25 females were introduced into four exposure tubes, each containing insecticide-treated filter paper. For alpha-cypermethrin, initial tests were conducted with the insecticide alone, followed by tests in combination with Piperonyl Butoxide (PBO), a synergist that inhibits enzyme activity, particularly targeting oxidases. This approach aimed to explore the role of these enzymes in pyrethroid resistance across the different species.

During the test, the number of mosquitoes immobilized or killed by contact with the insecticide was recorded at various time intervals during the one-hour exposure period (15, 20, 30, 45, and 60 min). Two control groups, of equivalent size, were also exposed to untreated filter papers to serve as a reference. After the exposure period, mosquitoes were transferred to observation tubes and maintained at 27 °C with a relative humidity of 75%. The mosquitoes were then provided with a 10% sugar solution, and mortality was evaluated 24 h later, following the guidelines of the WHO.

### Discrimination of twin species of the *Anopheles gambiae* complex

To distinguish the different species within the *Anopheles gambiae* s.l. complex, genomic DNA was extracted using the Molecular Biology extraction kit (Bio Basic, Toronto, Canada), following the manufacturer’s instructions. Then, a PCR known as SINE (Short Interspersed Nuclear Element) is performed according to the method described by Santolamazza et al. (2008) [17], using the primers F6 and R6, which are 20 base pairs and 21 base pairs in length, respectively (Table 2). This approach allows for the identification and differentiation of species within the *Anopheles gambiae* s.l. complex based on specific genetic markers.

**Table 2:** Primer sequences used for detection of *kdr* mutations and identification of *Anopheles gambiae* and *Anopheles coluzzii*

Target	Primer name	Nucleotide sequences (5′-3′)	References
*kdr* gene region	Agd1	ATAGATTCCCCGACCATG	[18]
	Agd2	ACAAGGATGATGAACC	
*L1014F*	Agd3:	AATTTGCATTACTTACGACA	
*L1014L*	Agd4	CTGTAGTGATAGGAAATTTA’	
*L1014S*	Agd5	TTTGCATTACTTACGACTG	[19]
Species identification (*An. gambiae*, *An. coluzzii*)	F6	TCG CCT TAG ACC TTG CGT TA	[17]
	R6	CGC TTC AAG AAT TCG AGA TAC	

The thermal protocol of the PCR begins with an initial denaturation step at 94 °C for 3 min to separate the two DNA strands. This is followed by 35 amplification cycles, each consisting of three successive phases: a denaturation step at 94 °C for 30 s to separate the DNA strands again, an annealing step at 55 °C for 30 s allowing the primers to bind to the target sequences, and an elongation step at 72 °C for 30 s during which the polymerase synthesizes new DNA strands. Finally, a final elongation phase at 72 °C for 5 min is carried out to complete the extension of all amplified DNA fragments.

To visualize the target sequences after amplification, electrophoresis is performed on a 2% agarose gel. The expected sizes are: 479 bp for *Anopheles coluzzii*, 249 bp for *Anopheles gambiae* s.s., and 223 bp for *Anopheles arabiensis.*

### Molecular characterization of the different mutations

After WHO tube susceptibility tests conducted on the three mosquito species, molecular analyses targeting resistance mechanisms, particularly mutations in the *kdr* gene, were carried out on resistant individuals (i.e., those that survived 24 h after exposure to pyrethroid insecticides), randomly selected from *Aedes aegypti* and *Anopheles gambiae* populations. *Culex quinquefasciatus* was excluded from these molecular investigations, as it is primarily involved in the transmission of diseases for which Benin is no longer considered endemic.

### Genotyping of *L1014F *and *L1014S *mutations of the *kdr* gene in *Anopheles gambiae s.l*.

The search for the *L1014F* mutation of the *kdr* gene, which is associated with resistance to pyrethroids and organochlorines, is performed by amplifying *Anopheles* DNA according to the protocol [18]. The primers AgD1, AgD2, AgD3, and AgD4 (Table 2) are used for amplifying the sequence of interest. The primer pair AgD1/AgD2 flanks the mutation gene *kdr*, amplifying a 293 bp product as a control. AgD3 binds only to the resistant *kdr* allele, and when paired with AgD1, it amplifies a 195 bp fragment if the mutation is present in the individual. The AgD4/AgD2 pair binds only to the sensitive portion of the gene, amplifying a 137 bp fragment. Samples that did not present the *L1014F* mutation or the homozygous sensitive allele were re-processed using a new primer, AgD5, which binds to the allele carrying the *L1014S* resistance mutation and amplifies a 195 bp fragment [19]. The reaction mixture for both PCR tests remains the same, with the only difference being the primer used for the resistance mutation. The PCR products are migrated on a 2% agarose gel stained with ethidium bromide and visualized under UV light.

### Genotyping of mutations *V1016I, S989P *and* F1534C* in *Aedes* mosquitoes

*Aedes* mosquitoes exhibiting phenotypic resistance to permethrin, deltamethrin, and alpha-cypermethrin were selected for genotyping of the three *kdr* mutations (*F1534C, V1016I,* and *S989P*) using the PCR-AS (allele-specific PCR) method. The detection of the V1016I mutation was carried out according to the protocol of Martins et al. [20], while the mutations S989P and F1534C were identified following the method of Li et al. [21].Modifications were made as in the work of Sombié et al. (2019) [22]. Each PCR reaction was performed in a total volume of 12.5 μl.

For the PCR reaction of the *V1016I* mutation, the reaction mixture included 1 μl of target DNA; 2.5 μl of primer mix Val1016f (0.125 μM), Iso1016f (0.125 μM), Iso1016r (0.25 μM); 2.75 μl of sterile water; and 6.25 μl of a MgCl2 10X buffer mixture, dNTP, and Taq polymerase (Thermo Scientific). The PCR thermal program included an initial denaturation at 95 °C for 10 min, followed by 35 cycles of: denaturation at 95 °C for 30 s, hybridization at 60 °C for 1 min, extension at 72 °C for 30 s, and a final extension at 72 °C for 5 min. After amplification, PCR products were loaded into wells and subjected to electrophoresis on a 2% agarose gel in TAE buffer. DNA fragment detection was performed by ethidium bromide staining and visualization under UV light.

Genotyping of the F1534C mutation required two sets of PCR reactions: the first used a primer mix of M3-For, M3-Rev, and M3-F, while the second used a primer mix of M3-For, M3-Rev, and M3-C to detect the 1534F and 1534C alleles, respectively. The volume of each PCR reaction was 12.5 µL, containing 1 µL of target DNA, 2.5 µL of primer mix (final concentration of 0.5 µM for each primer), 2.75 µL of sterile water, and 6.25 µL of a mixture of dNTP, Taq polymerase (Thermo Fisher Scientific). PCR cycling conditions were: initial denaturation for 10 min at 95 °C, followed by 35 cycles of: denaturation at 95 °C for 30 s, hybridization at 60 °C for 30 s, extension at 72 °C for 30 s, and a final elongation at 72 °C for 5 min.

For the *S989P* mutation genotyping, the reaction mixture remains the same, except for the different primers (Table 3). The thermal program for amplification is almost the same, except for the annealing temperature, which is set at 62 °C. Electrophoresis was performed on a 1.5% agarose gel for both mutations in TAE buffer and stained with an ethidium bromide solution for UV visualization. The size of the amplified products for the *V1016I* mutation is 98 bp for wild-type alleles and 78 bp for mutant alleles [20]. For the *F1534C* mutation, the size of the products is 284 bp for the wild-type allele and 240 bp for the mutant allele [21].

**Table 3:** Primer sequences used for the detection of *kdr* mutations *V1016I, F1534C*, and *S989P* in *Aedes aegypti*

*kdr* Mutations	Primer sequences	References
*V1016I*	Iso1016f: 5′-GCG GGC ACA AAT TGT TTC CCA CCC GCA CTG A-3′ Val1016f: 5′-GCG GGC AGG GCG GGG GCG GGG CCA CAA ATT GTT TCC CAC CCG CAC CGG-3′ Reverse primer: 5′-GGA TGA ACC GAA ATT GGA CAA AAG C-3′	[20]
*F1534C*	M3-For GGAGAACTACACGTGGGAGAAC M3-Rev CGCCACTGAAATTGAGAATAGC M3-F GCGTGAAGAACGACCCGA M3-C GCGTGAAGAACGACCCGC	[21]
*S989P*	M1-For AATGATATTAACAAAATTGCGC M2-Rev GCACGCCTCTAATATTGATGC M1-S GCGGCGAGTGGATCGAAT M1-P GCGGCGAGTGGATCGAAC	[21]

### Allelic frequencies of resistance genes

To calculate the allelic frequencies of the *kdr* gene for each mutation, the different expected genotypes in the population are: AA, Aa, and aa. The allelic frequency is calculated using the following formula:

$$f\left( A \right) \, = \, \left( {2N_{AA} + \, N_{Aa} } \right) \, / \, 2\left( {N_{AA} + \, N_{Aa} + \, N_{aa} } \right)$$

where N_AA_ is the number of individuals homozygous for allele A (carrying two copies of allele A), N_Aa_ is the number of heterozygous individuals (carrying one copy of allele A and one copy of allele a), N_aa_ is the number of individuals homozygous for allele a (carrying two copies of allele a).

This formula was used to estimate the frequency of the different mutations of the *kdr* gene studied [23].

### Statistical analysis and interpretation

The interpretation of the mortality rates observed during the tube susceptibility tests is done in accordance with the recommendations of the WHO [24]. A mosquito population was considered susceptible when the mortality rate was between 98 and 100%. A mortality rate between 90 and 97% suggested potential resistance, while a mortality rate below 90% indicated confirmed resistance. The Chi-square (χ^2^) test was performed to assess the significance of the difference between mortality rates.

## Results

### WHO susceptibility test

A total of 1775 mosquitoes were tested, including 592 *Aedes*, 596 *Culex*, and 587 *Anopheles* (Table 4), exposed to different insecticides according to the protocols established by the WHO [24]. These tests allowed for the evaluation of mortality rates induced by exposure to various classes of insecticides.

**Table 4:** Number of individuals tested by species and by insecticide

	*Aedes aegypti*	*Culex quinquefasciatus*	*Anopheles gambiae*
Tested insecticides	Tested number		
Bendiocarb 0.1%	97	99	96
Pirimiphos methyl 0.25%	99	100	101
Deltamethrine 0.05%	99	97	95
Permethrine 0.75%	100	100	100
Alphacypermethrine 0.5%	98	100	98
Alphacypermethrine 0.5% + PBO	99	100	97
Total	592	596	587

### Assessment of phenotypic insecticide resistance in *Aedes aegypti*

**Fig. 5:**
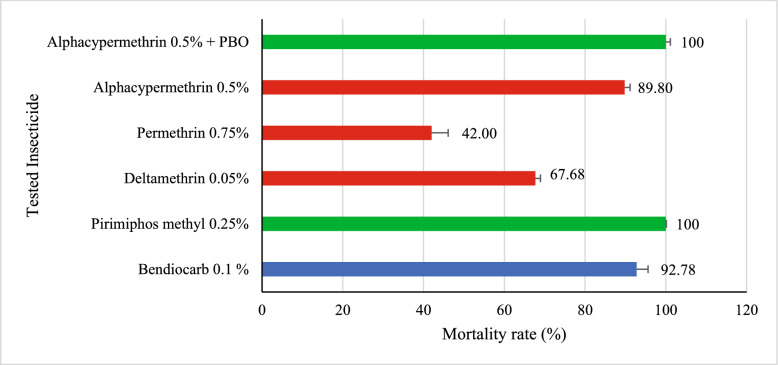
Susceptibility status of *Aedes aegypti*

For *Aedes*, the results obtained after exposure to bendiocarb indicate suspected resistance of this species to this insecticide. In contrast, mosquitoes were found to be completely susceptible to pirimiphos methyl, confirming the effectiveness of this molecule against *Aedes aegypti*. Regarding deltamethrin and permethrin, the results revealed a strong resistance (mortality < 90%) of the species to these two pyrethroids, with respective mortality rates of 67.68% (CI [57.95–76.08]) and 42% (CI [32.80–51.79]). Exposure to alpha-cypermethrin alone revealed resistance, with a mortality rate of 89.79% (95% CI [82.23–94.36]). However, when this insecticide was combined with PBO (piperonyl butoxide), complete mortality of mosquitoes was observed 100% (95% CI [94.50–99.82]), indicating a restoration of susceptibility. The difference in mortality rates between the two conditions was statistically significant (χ^2^ = 8.63, *p* = 0.0033), suggesting that the detected resistance may be associated with enzymatic mechanisms, particularly involving oxidases or esterases (Fig. 5).

### Assessment of phenotypic insecticide resistance in *Culex quinquefasciatus*

**Fig. 6:**
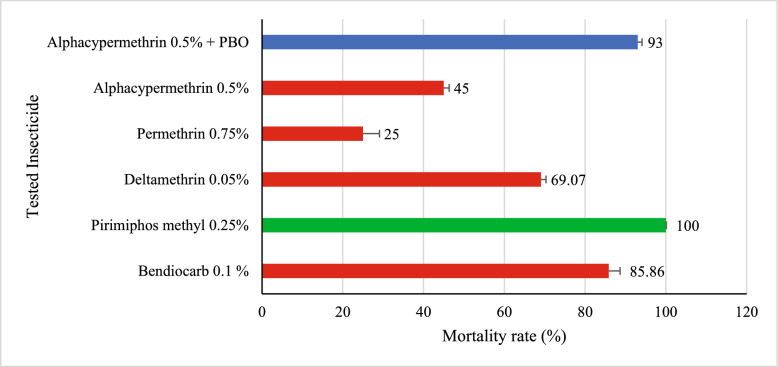
Susceptibility status of *Culex quinquefasciatus*

In *Culex quinquefasciatus*, susceptibility tests (Fig. 6) revealed low efficacy of bendiocarb (85.86% mortality), deltamethrin (69.07%), as well as alpha-cypermethrin and permethrin (25%), indicating notable resistance of the population to these different classes of insecticides. A 100% mortality rate was recorded following exposure to pirimiphos-methyl, indicating full susceptibility of *Aedes* to this organophosphorus insecticide. In contrast, exposure to alpha-cypermethrin alone resulted in a mortality rate of 45% (CI [35.61–54.76]), reflecting a high level of resistance. The addition of the synergist PBO significantly increased mortality to 93% (CI [86.25–96.57]), although this did not reach the threshold for full susceptibility. This difference is statistically significant (χ^2^ = 51.636; *p* < 0.0001) and suggests metabolically based resistance, partially overcome by PBO.

### Assessment of phenotypic insecticide resistance in *Anopheles gambiae*

**Fig. 7:**
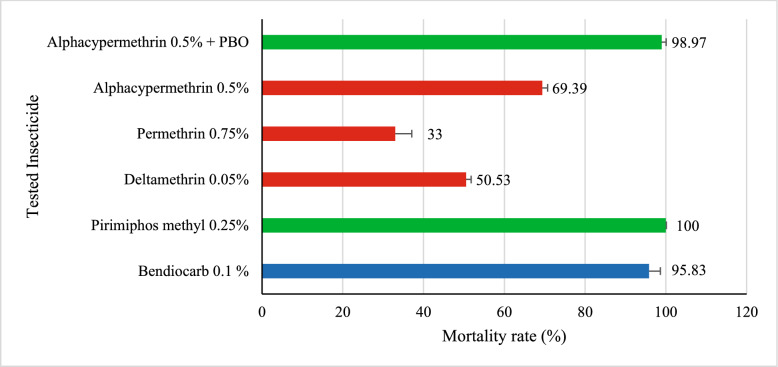
Susceptibility Status of *Anopheles gambiae s.l*

In *Anopheles gambiae*, susceptibility tests (Fig. 7) revealed suspected resistance to bendiocarb, with a mortality rate of 95.83% (CI [89.77–98.37]). In contrast, pirimiphos-methyl induced complete mortality in the tested mosquitoes (100%, CI [96.34–100]), indicating full susceptibility of the population to this organophosphate insecticide.

Regarding the evaluated pyrethroids: deltamethrin, permethrin, and alpha-cypermethrin, the results revealed high levels of resistance, as reflected by the respective mortality rates of 50.53% (CI [40.65–60.36]), 33% (CI [24.56–42.69]), and 69.39% (CI [59.68–77.64]). However, the addition of the synergist PBO to alpha-cypermethrin resulted in a significantly higher mortality rate of 98.97% (CI = [92.79–99.43]). This improvement was statistically significant compared to alpha-cypermethrin alone (χ^2^ = 29.731; *p* < 0.0001).

### Detection of mutations and allelic frequencies in the port of Cotonou *L1014F* and *L1014S*

#### Mutations in *Anopheles gambiae s.l.*

A total of 144 *Anopheles gambiae* s.l. specimens were analyzed using SINE-PCR, identifying 38 *Anopheles gambiae* s.s. and 106 *Anopheles coluzzii*. These specimens were subsequently subjected to Diagnostic PCR to detect the presence of *kdr* mutations *L1014F* and *L1014S*. The results revealed the exclusive presence of the *L1014F* mutation in both species. The allelic frequencies of the resistance allele *f(F)* were high, reaching 0.84 in *An. gambiae* s.s. and 0.92 in *An. coluzzii*. No homozygous susceptible individuals (*L1014L*) were detected in either species, suggesting a strong fixation of the resistance allele in these populations.

As for the *L1014S* mutation, it was observed only in *Anopheles gambiae* s.s., where it was associated with resistance in the heterozygous state. Heterozygous individuals carrying this mutation were found at a low allelic frequency of *f(S)* = 0.052. However, this mutation was not detected in *Anopheles coluzzii* (Table 5).

**Table 5:** Allelic frequencies of *L1014S* and *L1014F* mutations in *Anopheles gambiae* and *Anopheles coluzzii*

Mutations	Number tested	*Anopheles gambiae s.s*				Number tested	*Anopheles coluzzii*			
		Genotypes			Freq		Genotypes			Freq
		RR	RS	SS			RR	RS	SS	
L1014F	38	26	12	0	0.8421	106	91	15	0	0.9292
L1014S		0	4	34	0.052		0	0	106	0

*Freq* Frequency

#### *kdr* mutations V1016I, S989P and F1534C in *Aedes aegypti*

In the species *Aedes aegypti*, three *kdr* mutations (*V1016I, S989P* and *F1534C*) were investigated. A total of 186 individuals were subjected to allele-specific PCR, and all three mutations were detected with allelic frequencies of *f(C)* = 0.72, *f(I)* = 0.19 and *f(P)* = 0.63 for *F1534C, V1016I,* and *S989P*, respectively (Table 6).

**Table 6:** Allelic frequencies of *F1534C, S989P* and *V1016I* mutations in *Aedes aegypti*

Number of mosquitoes tested	Genotype and number of specimens									Allelic frequencies		
	Mutation *F1534C*			Mutation *V1016I*			Mutation *S989P*					
	F/F	F/C	C/C	V/V	V/I	I/I	S/S	S/P	P/P	*f (C)*	*f (I)*	*f (P)*
186	26	49	111	129	42	15	21	93	72	0.7284	0.1935	0.637

*F/F*  Homozygous for Phenylalanine, *F/C*  Heterozygous: one Phenylalanine and one Cysteine, *C/C*  Homozygous for Cysteine, *V/V*  Homozygous for Valine, *V/I*  Heterozygous: one Valine and one Isoleucine, *I/I*  Homozygous for Isoleucine, *S/S*  Homozygous for Serine, *S/P*  Heterozygous: one Serine and one Proline, *P/P*  Homozygous for Proline

**Fig. 8:**
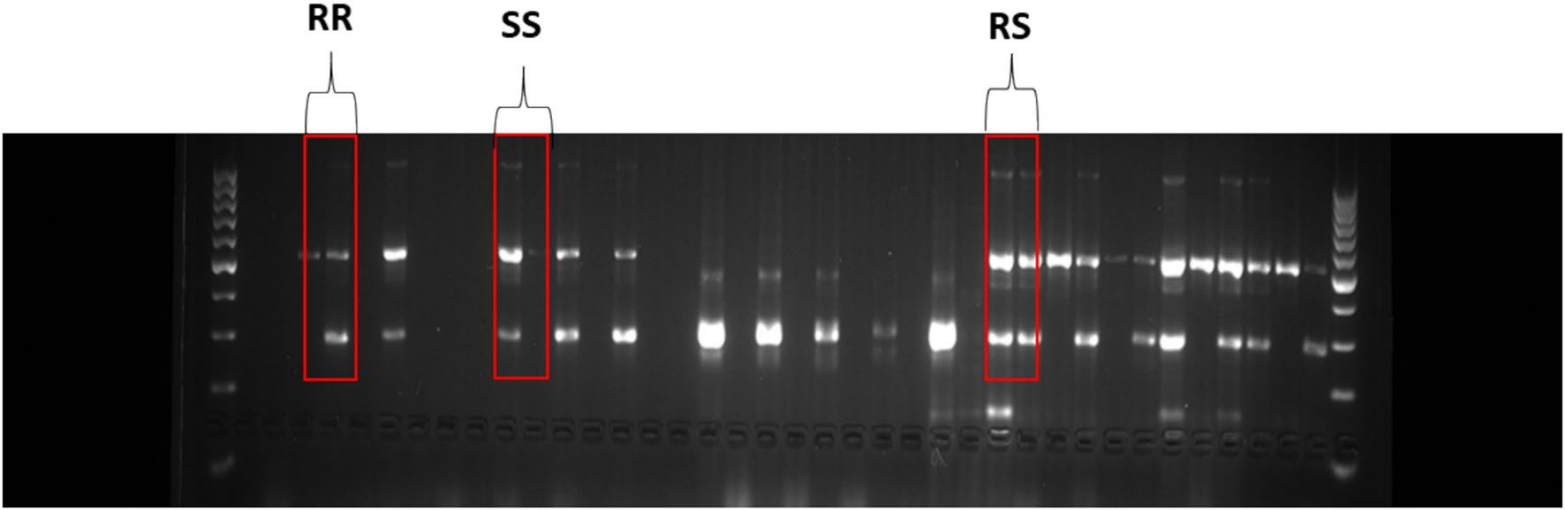
Agarose gel image for the *F1534C* mutation after amplicon migration

Figure 8 shows the band sizes obtained after migration for the *F1534C* mutation.

The image remains the same for the *V1016I* and *S989P* mutations; only the band sizes differ.

## Discussion

The World Health Organization (WHO) recommends monitoring insecticide resistance in all countries implementing vector-borne disease control programs. Over the years, most countries have detected resistance to several classes of insecticides, particularly pyrethroids. The results of this study reveal high phenotypic resistance in Anopheles gambiae to several classes of insecticides, particularly pyrethroids, which are the most commonly used class in vector control interventions [25]. The low efficacy of pyrethroids poses a significant threat to vector control programs, including indoor residual spraying (IRS) campaigns and the distribution of insecticide-treated nets, as these compounds are valued for their selective toxicity and repellent effect [24]. This situation is especially concerning as it may undermine the progress made in malaria control in the country, given the lack of alternative, less toxic insecticides. However, the combination of alpha-cypermethrin with the enzyme synergist piperonyl butoxide (PBO) achieved a mortality rate of 97.94%, indicating a restoration of efficacy. This suggests a major involvement of metabolic resistance mechanisms, particularly the overactivity of monooxygenases (P450s) and/or esterases, as previously documented in other studies conducted in Benin and West Africa [26, 27]. These findings support the use of next-generation nets combining pyrethroids and PBO in areas with high resistance to fight malaria, or alternatively, the use of pirimiphos-methyl in IRS campaigns. The suspected resistance to bendiocarb may reflect a geographic spread of resistance to this insecticide [26], potentially linked to mosquito population movements or increased selection pressure due to the prolonged use of this compound in urban areas. This is concerning, as previous studies had reported susceptibility of Anopheles populations to bendiocarb only a few years ago [28].

Regarding *Aedes aegypti*, in this study, alpha-cypermethrin alone did not demonstrate significant efficacy, but its effectiveness markedly increased when combined with the synergist PBO, suggesting a probable role of metabolic resistance mechanisms, particularly cytochrome P450 monooxygenases [29]. These results contrast with those reported by Konkon et al. (2023) [30], who observed susceptibility of *Aedes aegypti* to alpha-cypermethrin alone in Porto-Novo, southeastern Benin. This discrepancy could be explained by distinct selective pressures between the study areas and potential biases in the testing procedures. Further studies are needed to assess the role of metabolic resistance. Furthermore, although certain formulations such as pirimiphos-methyl or alpha-cypermethrin combined with PBO have shown good efficacy against *Aedes aegypti*, this species largely escapes the impact of indoor residual spraying (IRS) due to its exophilic behavior and diurnal activity [30]. Therefore, effective control of this species requires targeted strategies, such as spatial spraying of insecticides and larvicidal treatment of breeding sites, using the aforementioned compounds in appropriate formulations.

Although *Culex quinquefasciatus* has been less studied due to its limited involvement in the transmission of currently prioritized public health diseases in Benin, its high nuisance potential and the sporadic persistence of lymphatic filariasis cases justify rigorous monitoring of its insecticide susceptibility. Indeed, this species exhibits widespread resistance to many public health insecticides, particularly pyrethroids [24]. This situation may be linked to its adaptation to urban environments, where larval habitats are often polluted with organic waste and household chemicals, creating selective pressure that favors the emergence and maintenance of resistance mechanisms [31]. Moreover, several studies have shown that resistance to pyrethroids in *Culex* is longstanding, having developed well before the massive deployment of these compounds for malaria vector control, which significantly complicates efforts to manage this species [32]. However, since *Culex* is an endophilic species, pirimiphos-methyl, which has demonstrated efficacy against this mosquito, could be used in indoor residual spraying (IRS) to significantly reduce its nuisance.

The *L1014S* mutation, previously reported in malaria vectors in West Africa and in Benin—particularly in the northern and central regions—was first identified about a decade ago in *Anopheles arabiensis* [33]. More recently, it has been detected in *Anopheles gambiae s.s.* populations from central and northern Benin, as reported in studies by Hessou-Djossou et al. (2024) [34]. The detection of this mutation challenges the previously accepted geographical distribution, which assumed it was limited to East Africa. However, it is now also found in neighboring countries such as Togo [13] and Burkina Faso [35].

Our study revealed the presence of the *L1014S* mutation in heterozygous *Anopheles gambiae s.s.* specimens in southern Benin, albeit at a low allelic frequency in the population. This observation may reflect sporadic migration of individuals carrying the mutation into this new area, thereby illustrating a progressive spread of the *L1014S* mutation within Benin. It is therefore necessary to expand this investigation to the entire southern region to assess the full extent of its distribution.

In contrast, the *L1014F* mutation displays high allelic frequencies in both species of the *Anopheles gambiae* complex, as reported historically [36–38]. These elevated frequencies suggest strong selective pressure in favor of the resistance allele, leading to near-fixation in *An. gambiae s.l.* populations. This level of resistance represents a serious threat to vector control programs that rely on insecticides targeting sodium channels, such as pyrethroids.

This molecular study of mutations in *Aedes* has revealed the presence of three major mutations associated with pyrethroid resistance in *Aedes aegypti*: *F1534C, V1016I,* and *S989P*. These mutations affect the voltage-gated sodium channel gene (*vgsc*), which is the primary target of pyrethroids, and are known to confer knockdown resistance (*kdr*) in mosquitoes.

The *F1534C* mutation was the most frequent in the tested population, with an allele frequency of 0.72, and a majority of individuals being homozygous resistant (C/C, n = 112). This mutation is well documented for conferring moderate resistance to type I pyrethroids (such as permethrin), and it may also enhance the resistance conferred by other kdr mutations when co-occurring [39]. The high prevalence observed suggests intense selection pressure likely due to frequent pyrethroid use in the study area.

The *V1016I* mutation, detected for the first time in Benin, showed a lower allele frequency *f(I)* = 0.20, with most individuals being homozygous susceptible (V/V). However, 59 individuals carried the mutant allele (V/I or I/I), indicating a notable presence of emerging resistance. This mutation is associated with increased resistance to type II pyrethroids (such as deltamethrin) and is frequently found in combination with *F1534C* [40, 41]*.* Its low frequency may reflect an early stage of spread or lower local selection pressure.

The S989P mutation showed an intermediate allele frequency *f(P)* = 0.63, with a relatively balanced distribution of S/S, S/P, and P/P genotypes. It is often associated with the *V1016I* mutation, and their co-occurrence enhances resistance to pyrethroids [42, 43]. A thorough understanding of the genetic combinations involved in resistance is therefore essential to guide insecticide selection and adapt control strategies. The high frequency of S989P, even in the absence of a high *V1016I* prevalence, could suggest differentiated selection dynamics or that this mutation has been established for a longer time.

## Conclusion

This study on insecticide resistance among the main vectors in the Port of Cotonou highlights resistance to pyrethroids in *Anopheles gambiae*, *Aedes aegypti*, and *Culex quinquefasciatus*. However, the effectiveness of alpha-cypermethrin combined with PBO against *Anopheles* and *Aedes* suggests that enzymatic mechanisms, such as esterases and monooxygenases, play a major role in this resistance. Pirimiphos-methyl, which remains effective against all species, emerges as a valuable option for vector control strategies. Furthermore, the presence of the *L1014S* mutation in *Anopheles gambiae* and the *V1016I* mutation in *Aedes aegypti* reflects a broader geographic spread of these mutations, which could compromise the effectiveness of vector control programs targeting both species. It is therefore crucial to continue resistance monitoring, adopt appropriate management strategies, and explore the use of alternative compounds to ensure the effectiveness of vector control efforts in Benin.

## Data Availability

The datasets used and/or analyzed during the current study are available from the corresponding author on reasonable request.
